# Impact of GnRH agonist triggering and intensive luteal steroid support on live-birth rates and ovarian hyperstimulation syndrome: a retrospective cohort study

**DOI:** 10.1186/1757-2215-6-93

**Published:** 2013-12-26

**Authors:** Stamatina Iliodromiti, Vuong Thi Ngoc Lan, Ho Manh Tuong, Phung Huy Tuan, Peter Humaidan, Scott M Nelson

**Affiliations:** 1 Maternal and Reproductive Medicine, School of Medicine, University of Glasgow, Glasgow, UK; 2 Department of Obstetrics and Gynecology, University of Medicine and Pharmacy HCMC, Ho Chi Minh City, Vietnam; 3 Research Center for Genetics and Reproductive Health (CGRH), School of Medicine, Vietnam National University HCMC, Ho Chi Minh City, Vietnam; 4 IVFAS, An Sinh Hospital, Ho Chi Minh City, Vietnam; 5 The Fertility Clinic, Skive Regional Hospital, Denmark and Aarhus University, Faculty of Health, Aarhus, Denmark

**Keywords:** GnRH agonist, GnRH antagonist, Luteal support, Live-birth, Ovarian hyperstimulation syndrome

## Abstract

**Background:**

Conventional luteal support packages are inadequate to facilitate a fresh transfer after GnRH agonist (GnRHa) trigger in patients at high risk of developing ovarian hyperstimulation syndrome (OHSS). By providing intensive luteal-phase support with oestradiol and progesterone satisfactory implantation rates can be sustained. The objective of this study was to assess the live-birth rate and incidence of OHSS after GnRHa trigger and intensive luteal steroid support compared to traditional hCG trigger and conventional luteal support in OHSS high risk Asian patients.

**Methods:**

We conducted a retrospective cohort study of 363 women exposed to GnRHa triggering with intensive luteal support compared with 257 women exposed to conventional hCG triggering. Women at risk of OHSS were defined by ovarian response ≥15 follicles ≥12 mm on the day of the trigger.

**Results:**

Live-birth rates were similar in both groups GnRHa vs hCG; 29.8% vs 29.2% (p = 0.69). One late onset severe OHSS case was observed in the GnRHa trigger group (0.3%) compared to 18 cases (7%) after hCG trigger.

**Conclusions:**

GnRHa trigger combined with intensive luteal steroid support in this group of OHSS high risk Asian patients can facilitate fresh embryo transfer, however, in contrast to previous reports the occurrence of late onset OHSS was not completely eliminated.

## Background

Gonadotrophin releasing hormone agonists (GnRHa) are highly effective in inducing an LH surge, with levels comparable to those observed during the spontaneous surge during normal menstrual cycles [1]. These characteristics have been exploited clinically, with avoidance of hCG trigger and adoption of GnRHa trigger in women at risk of ovarian hyperstimulation syndrome (OHSS) [2]. However, although a GnRHa induced LH surge is capable of inducing oocyte maturation, it is significantly shorter than that observed during natural cycle, leading to a compromised corpus luteal function [1,3]. Evidence for defective luteal function included the observation that GnRHa cycles have a shorter luteal phase and that the luteal steroid profile is reduced in both non-supplemented and supplemented in-vitro fertilisation (IVF) cycles, as compared to an hCG trigger [4]. The clinical impact of this defective corpus luteal function after a GnRHa trigger, is that when combined with standard luteal phase support, pregnancy rates are lower and miscarriage rates higher [5-7].

Recognition of these issues has prompted debate regarding the best strategy of luteal phase support to facilitate fresh embryo transfer in GnRHa triggered cycles. Two primary alternatives strategies have been suggested, with both dissociating the ovulation trigger from the luteal support. The first uses a modified luteal-phase support with a low dose of hCG administered after oocyte aspiration to replace the actions of early luteal LH to sustain implantation and endogenous luteal ovarian steroidogenesis [8-11]. The second avoids hCG and instead focuses on correcting the abnormal luteal steroid profile by providing intensive luteal-phase support with oestradiol and progesterone, only [12,13].

With respect to this second strategy Babayof et al. initially reported the use of intensive oestradiol and progesterone for luteal support in high-risk OHSS patients post GnRHa trigger [12]. In that pilot study (N = 15 in GnRHa group, N = 13 in hCG group) the luteal phase was supported by 50 mg/day of intramuscular progesterone, starting 36 h after oocyte retrieval and if serum progesterone concentration was below 40 nmol/l, the progesterone dose was doubled. In addition if the serum oestradiol concentration was below 200 pmol/l, oral oestrogen 4 mg/day was added. Only one woman in the GnRHa trigger group achieved a live birth (6.7%), as compared to two live births in the hCG group where 11 women received an embryo transfer (18%). Given these poor outcomes, Engmann et al. suggested a more aggressive luteal support in their RCT in which 30 women in the GnRHa trigger group were treated with 50 mg/day of intramuscular progesterone, but aimed to maintain levels above 20 ng/ml (63.6 nmol/l), while oestrogen was added by 0.3 mg transdermal patches every other day, and supplemented by a further 0.1 mg and/or oral micronized oestradiol 2 mg twice a day to maintain serum oestradiol levels above 200 pg/ml (734 pmol/l) [13]. This approach was associated with substantially better ongoing pregnancy rates (53.3%), equivalent to those observed in the hCG triggered control group (48.3%). Importantly no OHSS case was reported after GnRHa trigger versus 31% after hCG trigger. Shapiro and colleagues subsequently reported their experience of a similar enhanced luteal support strategy in 24 women aiming to maintain serum oestradiol levels above 200 pg/ml and progesterone above 15 ng/ml (47.7 pmol/l) and reported a 50% ongoing pregnancy rate [14]. Imbar et al. in their study of 70 women used a strategy of just 50 mg/day of intramuscular progesterone plus 6 mg of oestradiol, reported an overall live birth of 27.1% [15]. Lastly, Orvieto reported in 67 women that use of either 100 mg intramuscular progesterone or 200 mg twice a day vaginal progesterone combined with 6 mg oral micronized 17-β-oestradiol was associated with a low clinical pregnancy rate of 14.9% [16], comparable to pregnancy rates previously published after GnRHa trigger without intensive luteal support (Orvieto et al., 2006). No OHSS case was seen after GnRHa trigger and intensive luteal support in any of the above-mentioned trials.

Whether the wide variability in clinical outcomes after GnRHa trigger and luteal steroid support, only, reflects the differing steroid regimens or whether ethnical differences may also play a role is unknown. However, it has been suggested that follicular response and the associated peak oestradiol at the time of GnRHa trigger may influence treatment outcomes [17]. Specifically a peak oestradiol of <4,000 pg/ml was associated with a lower ongoing pregnancy rate, as compared to oestradiol >4,000 pg/ml (ongoing pregnancy rate 33.2% vs 44.9%; p = 0.02) [17]. However, classically an excessive ovarian response has been associated with a lower live birth rate [18] and in this aspect supra-physiological oestradiol levels have been shown to have a deleterious effect on embryo adhesion and a direct toxic effect on the embryo [19].

Given the lack of consistency in the literature regarding intensive luteal phase support with steroids, only, after GnRHa trigger, we here report treatment outcomes in a Vietnamese population after GnRHa trigger as compared to controls exposed to an hCG trigger and conventional luteal support.

## Methods

### Study subjects

**Figure 1:**
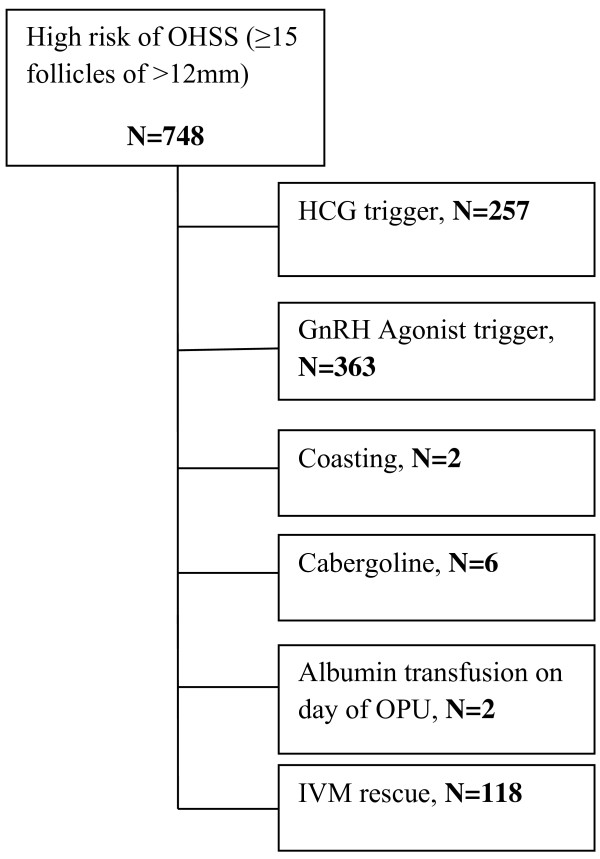
**Flow chart depicting the various protocols used for OHSS high risk patients in the unit during the study period from January 2010 to October 2012**. **(OPU: ovum pick up, IVM: in vitro maturation).**

This retrospective analysis summarizes the experience from a large Vietnamese fertility centre using a GnRHa protocol to trigger final oocyte maturation and an intensive luteal support package and compares it with the use of the conventional hCG trigger protocol, to facilitate a fresh transfer in women deemed at high risk of developing OHSS. All the patients undergoing the GnRHa trigger protocol attended the clinic from January 2010 to October 2012. The criteria used for identifying women with a high risk of developing OHSS included actual ovarian response [20]; specifically ≥15 follicles measuring ≥12 mm in size on the day of the trigger. The unexposed group included all the patients fulfilling the same response criteria during the same period receiving conventional hCG trigger. From January 2012 to October 2012, the GnRHa package had fully replaced the conventional hCG protocol for patients deemed at high risk of OHSS, therefore there were no patients with excessive ovarian response who received the hCG conventional trigger protocol during this period. The allocation of the patients to either protocol during the transitional period was based on clinicians’ preference. All cycles which used alternative techniques such as ‘coasting’, or cabergoline therapy to minimize the risk of OHSS were excluded from the study. Figure 1 shows a flowchart of the selection of the patients to each protocol per year of treatment. Oocyte donation cycles were also excluded. The clinical and laboratory personnel did not change over the study period.

### IVF treatment protocol

A GnRH antagonist protocol was used as the primary mode of stimulation co-treatment with the gonadotrophin dose adjusted according to patients’ baseline characteristics. The standard starting dose of recombinant follicle stimulating hormone (rFSH, Puregon MSD) was 150 IU per day for women under 36 years of age. Gonadotrophin doses were, however, modified based on age, body mass index (BMI), presence of polycystic ovary syndrome (PCOS), antral follicle count (AFC), anti-müllerian hormone (AMH) and previous history of OHSS so that starting FSH doses ranged from 100 IU to 225 IU. Stimulation started on day 2 or 3 of the cycle and a GnRH antagonist was added on day 5 of stimulation (day 6 of the cycle). Pelvic ultrasound was performed on stimulation day 6, with adjustment of the gonadotrophin according to ovarian response. Routine ultrasound and endocrine monitoring were initiated thereafter, with triggering when three follicles were ≥17 mm in size. The decision to use a GnRHa trigger was made in women with excessive follicular response (≥15 follicles ≥12 mm). GnRHa was administered at least 8 hours following the last GnRH antagonist injection. No upper cut-off limit was used, with all women proceeding to trigger in the GnRHa group. For the GnRHa trigger a subcutaneous injection of 0.2 mg Triptorelin (Ipsen Beaufour, France) was used. Oocyte retrieval was performed 34 to 36 hours following GnRHa administration. Fertilization was undertaken using standard protocols and all embryos were cultured for two days, before being either transferred or cryopreserved. Three embryos were routinely transferred on day two of culture.

The luteal support strategy utilised was intramuscular progesterone 50 mg once per day (Progesterone BP 25 mg, Rotexmedica, Germany) plus vaginal progesterone twice daily (Crinone 90 mg, Merck Serono), and oestradiol valerate 6 mg daily (Progynova 2 mg, Bayer Schering) administered from the day of egg retrieval until menstruation or 7 weeks of gestation. No additional monitoring for adjustment of the steroidal dosage was performed.

In the hCG control group, stimulation was achieved similarly with rFSH from day 2 to 3 combined with a GnRH antagonist added on day 5 of stimulation. On achieving 3 follicles ≥17 mm in size, 250 μg rhCG (Ovitrelle, Merck Serono) was administered subcutaneously to induce oocyte maturation and egg retrieval followed 34–36 hours later. Three embryos were transferred two days after routine embryo culture. Luteal support commenced on the day of egg retrieval consisting of twice daily vaginal progesterone (Crinone 8%, Merck Serono) until the day of the pregnancy test (14 days post embryo transfer).

### Ethics approval

Full ethics committee approval was not required due to the retrospective nature of the study and the anonymised handling of data.

### Outcomes and statistical analysis

The two primary outcomes of this retrospective cohort study were the live birth rate and the incidence of severe OHSS. The Royal College of Obstetricians and Gynaecologist (RCOG) classification of OHSS was used with severe OHSS requiring hospitalisation, and mild or moderate OHSS managed on an outpatient basis [21]. Ongoing pregnancy rate was defined as continuing pregnancy after 24 weeks. Positive pregnancy test was defined as a positive serum hCG on day 16 post oocyte retrieval. Clinical pregnancy was defined by the presence of at least one fetal heart on an 8 week ultrasound. Miscarriage was defined as pregnancy loss before 24 weeks gestation. The implantation rate was calculated by dividing the number of fetal hearts detected at 8 weeks’ gestation ultrasound by the number of embryos transferred. Multiple pregnancy rate was defined as the proportion of multiple pregnancies at 8 weeks’ gestation scan divided by the number of clinical pregnancies at this gestation. Live birth, positive pregnancy test, clinical, ongoing pregnancy and miscarriage rates are reported per cycle started unless otherwise stated. The effect of peak oestradiol (E2) and LH on the pregnancy rate of patients undergoing the GnRHa trigger protocol was also evaluated.

Statistical analysis was performed using Stata/SE (version 12.1, Stata Corp, USA) and data are presented as mean ± SD (standard deviation) when they have a Gaussian distribution or median (25^th^-75^th^ range) when they are not normally distributed. Non parametric test (Mann Whitney U) was used for not normally distributed numerical variables and chi-squared or Fisher’s exact test for categorical variables. Logistic regression was performed with adjustment for confounders. P < 0.05 was considered statistically significant for all analyses.

## Results

### Demographic data and cancellations

Table 1 shows the baseline patients characteristics of the patients undergoing either GnRHa trigger followed by the intense luteal support package or the traditional hCG trigger protocol. Patients were in general young, slim and had high ovarian reserve indices consistent with an increased risk of an excessive ovarian response and development of OHSS. Furthermore 8.3% of the patients in the GnRHa group had previously experienced severe OHSS compared with 1.6% in the control group. In the GnRHa group 11.2% of the cycles (41 cycles out of 363) were cancelled due to thin endometrium < 6 mm (n = 28), personal reasons (n = 11) or early signs of OHSS (n = 2) such as bloating and epigastric pain. In the hCG group 17% of the cycles (44 cycles out of 257) were cancelled due to either thin endometrium < 6 mm (n = 15), personal reasons (n = 7) or early signs of developing OHSS (n = 22).

**Table 1:** Baseline characteristics of the patients receiving either GnRHa trigger accompanied with intensive luteal steroid support or conventional hCG trigger for final follicular maturation

	**GnRHa trigger plus intensive luteal steroid support**	**Conventional hCG trigger**	**p**
Cycles (n)	363	257	
Age (years)	31 (28–34)	30 (28–34)	0.97
BMI (kg/m^2^)	20.4 (19.1-21.9)	20.3 (19.2-21.4)	0.59
History of OHSS (n,%)	30 (8.3%)	4 (1.6%)	<0.0001
AMH (ng/ml)	5.85 (4–8.2)	6.17 (4.1-9.2)	0.36
AFC	16 (12–24)	13 (10–17)	0.0001
Aetiology (n,%)			
Male	153 (42.1%)	153 (59.5%)	
PCOS	102 (28.1%)	45 (17.5%)	
Endometriosis	4 (1.1%)	5 (2%)	
Tubal disease	62 (17.1%)	50 (19.5%)	
Idiopathic	30 (8.3%)	4 (1.5%)	
Unclassified	12 (3.3%)		

Variables that are not normally distributed are expressed as median (25th-75th percentile).

### Oocytes, embryos and reproductive outcome

Table 2 shows the treatment outcomes. A large number of oocytes were collected with both protocols with appropriate rates of fertilization, development and embryos available for cryopreservation. The positive pregnancy rate was 36.9% per cycle, with an overall clinical pregnancy rate of 30% per cycle started and live-birth rate of 29.8% among the patients that underwent the GnRHa trigger, which were similar to those observed in the hCG group. All patients with an ongoing pregnancy after 24 weeks had a live-birth in the GnRHa group excluding one patient who moved abroad and for whom information on delivery status was not available. When the analysis was restricted to women who had an embryo transfer, there was still no difference in live-birth rate between the two groups (p = 0.8). The overall miscarriage rate was lower in the GnRHa group compared to the hCG group per cycle started and per embryo transfer. After adjustment for age, BMI and cause of infertility the risk of miscarriage was still reduced in the GnRHa group per cycle started (OR 0.41, 95% CI 0.23, 0.71) or if limited to those who had an embryo transfer (OR 0.38, 95% CI 0.22, 0.67).

**Table 2:** Reproductive outcomes of patients exposed to GnRHa trigger and intensive luteal steroid support versus patients exposed to conventional hCG trigger for final follicular maturation

	**GnRHa trigger plus intensive luteal steroid support (N = 363)**	**Conventional hCG trigger (N = 257)**	**p**
Total FSH (IU)	1,500 (1,300-1,800)	1,650 (1,200-2,200)	0.12
Peak Oestradiol (E2) (pg/ml)	11,850 (8,700-15,395)	8,598 (5,510-12,350)	0.0001
LH on day of trigger (IU)	1.95 (1.21-3.48)	2.14 (1.28-3.49)	0.46
Follicles >12 mm	20 (17–25)	18 (16–21)	0.0001
Oocytes	19 (15–24)	18 (15–22)	0.02
Embryos	11 (7–15)	10 (7–13)	0.14
Embryo transfers (ET) (n,% of total cycles)	322 (88.7%)	213 (83%)	0.038
Cancellations of ET due to early signs of OHSS (n,% of total cycles)	2 (0.55%)	22 (8.6%)	<0.0001
Embryos transferred	3	3	
Positive pregnancy test (n,%per cycle)	134 (36.9%)	112 (43.5%)	0.095
Clinical Pregnancy (n,%per cycle)	109 (30%)	77 (30%)	0.99
Implantation rate (n,%)	137/966 (14.2%)	106/639 (16.6%)	0.978
Miscarriages (n,% per cycle)	5 (1.4%)	15 (5.8%)	0.002
Live birth rate (n,% per cycle)	108 (29.8%)	75 (29.2%)	0.69
Multiple pregnancy rate (n,% of live-birth)	28 (25.9%)	29 (38.7%)	0.067
OHSS cases (severe) (n,% per cycle)	1 (0.3%)	18 (7%)	<0.0001

Variables that are not normally distributed are expressed as median (25th-75th percentile).

Outcome data are presented per cycle started unless otherwise stated.

### Endocrinology

The peak oestradiol (E2) was recorded for 348 cycles undergoing GnRH trigger for oocyte maturation. The median peak serum E2 level was 11,850 pg/ml (8,700-15,395 pg/ml). 27 patients (7.8%) had peak E2 < 4,000 pg/ml and 321 patients (92.2%) peak E2 ≥ 4,000 pg/ml. The median peak E2 among those having E2 < 4,000 pg/ml was 2,408 (1,751-3,070) and in those having E2 ≥ 4,000 pg/ml was 12,350 (9,361-16,000) pg/ml. The ongoing pregnancy rate in women having had E2 < 4,000 pg/ml was 48.2% (13/27), whereas in patients with E2 ≥ 4,000 pg/ml was 28.4%% (91/321) per cycle started (p = 0.03). This difference was maintained when the analysis was restricted to those with an embryo transfer (p = 0.04). However, the distribution of peak E2 among those that became pregnant did not differ from those that did not (p = 0.21). Similarly, in univariate regression analysis pre-trigger E2 levels were not associated with the chance of live-birth in women undergoing GnRHa trigger (OR = 0.99, 95% CI 0.99-1, p = 0.082). LH on the day of the trigger was measured in 257 women undergoing GnRH trigger for oocyte maturation. The median LH level was 1.95 IU/L (1.21-3.48 IU/L). Univariate regression analysis indicated that one unit higher LH on the day of the GnRHa trigger increased the odds for a live-birth by around 12% (OR = 1.12, 95% CI 1.002-1.25, p = 0.046). Among those women with high (E2 ≥ 4,000 pg/ml) and low peak E2 (E2 < 4,000 pg/ml), LH did not differ (median, 75^th^ centile 1.96 (1.21-3.52) v 1.68 (0.97-2.9), p = 0.7).

### OHSS

With respect to OHSS, there was one severe late OHSS case (0.3%) among the women that had GnRHa trigger for oocyte maturation as opposed to 18 cases (7%) among the women having received the traditional hCG trigger (p < 0.0001). After adjustment for age, BMI, peak oestradiol and follicle number the odds ratio for development of OHSS with GnRHa trigger was OR 0.02 (95%CI 0.003 - 0.18).

The single late onset OHSS case, which is the first case reported in the literature after GnRHa trigger and intensive luteal steroid support, only, occurred in a 30 year old patient with a BMI of 20.4 kg/m^2^ who sought fertility treatment for primary infertility due to a male factor. She had a high baseline serum AMH of 10.68 ng/ml and an AFC of 24. Stimulation was initialized on day 2 of her cycle with 100 IU of recombinant FSH due to the high ovarian reserve indices. On day 6 of the cycle, 0.25 mg of a GnRH antagonist (Orgalutran, MSD) was added. On day 8, a pelvic ultrasound showed that there was only one follicle > 17 mm in each ovary so stimulation continued and a pelvic ultrasound was repeated on day 10 showing an excessive follicular response (27 follicles >12 mm). The pre-trigger peak E2 was 18,959 pg/ml; 32 oocytes were collected and 24 embryos were cultured. After 2 days, three embryos were transferred as per the routine protocol of the clinic. Ten days following the embryo transfer, the patient started complaining of shortness of breath and the following day she was admitted to the hospital with severe epigastric pain, abdominal distension, dyspnoea, mild ascites, minimal pleural effusion, a positive pregnancy test and haematocrit of 45.3% (31.4% initially). Initial management included supportive care, strict fluid monitoring and thromboprophylaxis, however, while in hospital, the ascites and pleural effusion deteriorated, the abdominal circumference increased and the ovarian volumes also increased. Intravenous albumin was administered and paracentesis performed with initial drainage of 500 mls. An abdominal drain was left in situ with a further 300 mls of clear ascetic fluid drained over the next four days, with removal of the drain thereafter. She continued to improve clinically with supportive management and was discharged after ten days. An ongoing twin pregnancy was confirmed at scan which resulted in a delivery of two healthy babies at 38 weeks of gestation. Interestingly, there were two more cases in the GnRHa trigger group with early signs of OHSS (bloating), however the embryo transfer was cancelled and the patients did not require any further treatment or hospitalisation.

## Discussion

The application of GnRHa trigger in GnRH antagonist controlled cycles has provided a unique opportunity to minimize the risk of OHSS in controlled ovarian stimulation. However, recognition of the poor clinical outcomes when combined with conventional luteal support in autologous fresh cycles has prevented its routine adoption [22,23]. To overcome this deficiency a variety of intensive luteal steroid support strategies have been assessed, with variable results [13-15]. Herein we report that intensive luteal support with exogenous steroids in a Vietnamese population is capable of achieving live-birth rates comparable to those of a control group exposed to a conventional hCG trigger. However, in contrast to previous reports, GnRHa trigger with intensive luteal steroid support, only, does not completely eliminate OHSS as one patient developed late onset severe OHSS.

This is, to our knowledge, the largest retrospective cohort study that evaluates the live-birth rates after GnRHa trigger and intensive luteal steroid support, only. A previous study which reported promising clinical pregnancy outcomes with GnRHa trigger was debated due to the majority of the participants having PCOS [13]. It was suggested that as PCOS is known to be associated with high circulating endogenous LH levels, and that this may persists into the luteal phase [24], that potentially the endogenous LH was not completely suppressed, with residual LH supporting the function of corpus luteum and the implanting embryo [25]. In the current study, we did not observe any difference in outcomes relative to the cause of infertility, despite only 28% of the patients having PCOS. An almost similar size study without a control arm demonstrated good reproductive outcomes too after GnRHa trigger combined with intensive luteal steroid support, only [17], however, that study did not report live-birth, the ultimate goal of assisted conception.

The aggressive luteal steroid support protocol used after GnRHa trigger, was initially suggested by Engmann by combining intramuscular progesterone and daily E2 supplementation [13]; however, in the Vietnamese unit luteal phase monitoring of progesterone and E2 is not routinely performed as the centre covers a large catchment area and patients are unable to attend for regular blood follow up. Whether even better outcomes could have been achieved by close monitoring of the luteal phase and optimisation of the steroid profile is unclear, however, luteal phase serum oestradiol is often low in non-supplemented and supplemented cycles after GnRHa trigger [4]. Similarly, optimisation of progesterone levels may be essential as conception cycles (natural and stimulated) have a higher, tight range of mid-luteal progesterone levels than observed with just ovulation [26]. Furthermore, successful IVF pregnancies have a higher mid luteal progesterone concentrations than unsuccessful cycles [26]. Whether the route of administration contributed to our improved outcomes compared to those previously reported for the vaginal route is unclear [16]. Previous randomized controlled trials regarding route of progesterone administration have demonstrated equivalence in normal IVF cycles [27], however, whether this also applies to aggressive luteal support warrants further assessment.

Recently, in the context of GnRHa triggered cycles, it has been shown that a peak E2 ≥ 4,000 pg/mL in conjunction with a higher LH on the day of oocyte trigger is associated with a 44.9% ongoing pregnancy rate as opposed to 33.2% rate in women with a peak E2 < 4,000 pg/mL [17]. In our cohort of 363 women who had a similar protocol, these findings could not be reproduced and indeed the opposite was found as a peak E2 < 4,000 pg/mL was associated with a higher pregnancy rate as compared to those patients whose peak E2 exceeded 4,000 pg/mL. This disparity between ours and the previous analysis, may reflect the small number of women with a peak E2 < 4,000 pg/ml or that rather than peak E2 being critical, the endogenous LH level on the day of trigger may be important. The importance of the endogenous LH level around the time of trigger is strengthened by the recent observation that use of a dual trigger (GnRHa trigger and a bolus of hCG 1000 IU) for those women with a peak E2 < 4,000 pg/mL was associated with an improvement in live birth rate from 30.9% to 52.9% with only one case of mild OHSS reported [28]. Furthermore, patients with an E2 ≥ 4,000 pg/mL in the study by Kummer et al. had higher endogenous pre and post-trigger LH levels, which may have contributed to supporting the luteal phase [17]. That it reflects LH activity rather than peak E2 would also potentially be consistent with the lack of association between peak oestradiol and pregnancy rates in the present study, however in our population pre-trigger endogenous LH on the day of the trigger did not differ among those women having peak E2 ≥ 4,000 pg/mL and those with peak E2 < 4,000 pg/mL. This indicates that the suggested threshold of 4,000 pg/mL may not apply to the Asian population, consistent with previous reports suggesting that women of Asian origin have higher E2 for a given number of oocytes as compared to non- Asian women [29,30]. When the live-birth rates were stratified per peak E2 quartiles in the subgroup with a peak E2 ≥ 4,000 pg/mL, no differences were observed, further strengthening that peak E2 may not play a critical role in obtaining a live-birth rate after GnRHa trigger and intensive luteal support.

Importantly, this is the first study, to our knowledge, confirming that OHSS may still occur in a GnRHa trigger protocol even if the luteal support package does not include exogenous hCG. A previous study has suggested the possibility of this [31], however, the findings of this study have subsequently been disputed [32]. Albeit that the risk is very low with only one case of severe OHSS developing in the 322 fresh embryo transfers, previous studies assessing similar protocols did not report any cases of OHSS which may have reflected their limited power to detect this, given their smaller sample sizes (n = 15 [12] and n = 33 [13]) or a less aggressive approach with less embryos transferred which may not be clinically relevant to some countries [17]. In our series, a twin pregnancy had occurred in the case complicated with late onset severe OHSS, which exposed the patient to high concentrations of endogenous hCG, an established risk factor of late onset OHSS [33]. Furthermore, this single case of severe OHSS in our study group occurred in a young patient with excessive follicular response (27 follicles over 12 mm) and a peak E2 of 18,959 pg/ml. In retrospect, it can definitely be argued that a fresh embryo transfer should not have been performed. However, at present there are no established follicular cut off levels to cancel fresh embryo transfer with the intensive luteal steroid package after GnRHa trigger and the arbitrarily acceptable range of peak E2 resulting from trials in women from other ethnic backgrounds may not apply to the Asian population. Moreover, there were cases in our cohort with higher peak E2 and follicular response who had a successful embryo transfer and still achieved a live-birth without developing OHSS. Thus, our data suggest that the use of a GnRHa trigger combined with intensive luteal steroid support will minimise the occurrence of late onset OHSS, however does not completely eliminate it.

We acknowledge that the routine transfer of 3 embryos performed in the centre may have amplified the risk of OHSS in women already having a high risk of developing OHSS [34]; however, in Vietnam, the lack of legal regulations regarding the number of embryos transferred and the high cost of IVF allows this practise which is also further encouraged by the couples’ desire to have a multiple pregnancy during their first attempt. Undoubtedly, the transfer of three embryos is not advisable, let alone in women with excessive ovarian response, and the centre is changing its policy as a further reduction and close to elimination of the risk of OHSS could be achieved through single embryo transfer in this group of patients. However, for patients in whom the possibility of an OHSS condition is medically unacceptable, the way to proceed is segmentation of the cycle [35].

### Strengths and limitations

Although this study is the largest cohort study showing promising live-birth outcomes and a low OHSS risk following fresh embryo transfer after a GnRHa trigger combined with aggressive luteal steroid support in an Asian population, there are a number of limitations. The retrospective character of the study limits the ability to select patients and match groups for known confounders. However, both groups had similar baseline characteristics (e.g. age and BMI), known to affect the chance of pregnancy after fertility treatment [36]. We also included all the patients having excessive follicular response and either of two protocols within the duration of the study without applying further selective processes. Our reported pregnancy rate in this case series is lower than reported in other studies using the similar GnRHa protocol [13]. This may reflect the variation in success rates among different centres, countries and populations, so the use of a control arm in our study allows us to present comparative results. In addition, the luteal steroid support in our study was discontinued 7 weeks after a positive pregnancy test as opposed to 10 weeks in the study by Engmann et al. [13] which could have resulted in a higher miscarriage rate due to profound luteolysis after GnRHa trigger. However, so far there is no evidence suggesting the optimal duration of luteal support after GnRHa trigger. Moreover, the most profound luteal insufficiency is evident within the first 2 weeks after GnRHa triggering i.e. several weeks [37] before the luteoplacental shift takes place. Finally, the miscarriage rate reported by other studies using luteal support for 10 weeks [17] is not lower (7.9%) than that of the present study (1.6%). Obviously to draw firm conclusions regarding the optimal duration of the steroid luteal phase support after GnRHa trigger a RCT is warranted.

We appreciate that cancellation of fresh transfers within both arms due to concerns regarding triggering and development of OHSS will have reduced the overall ongoing pregnancy rate per cycle started, however, our analysis was performed on an intention to treat basis. We are also aware of the fact that the transfer of three embryos, until now routinely performed in the centre, has recently been shown not to improve the chance of live birth [34], but substantially increase the risk of OHSS [38] and other adverse perinatal outcomes [34]. Thus, the single OHSS case presented herein might potentially have been avoided with a single embryo transfer [38]. This study should not be considered as an attempt to encourage the routine transfer of 3 embryos in women with excessive follicular response by using the GnRHa trigger, but it provides additional information on the outcomes of the protocol in a non Caucasian population with different baseline and socioeconomic characteristics from those presented in previous studies.

The reasons for the discordance in miscarriage rates are not clear, and would warrant confirmation in randomised controlled trials comparing the two approaches. It can be debated that the discontinuation of the luteal support at the day of the pregnancy test at the hCG arm may have contributed to the higher miscarriage rate in this group. However, the current evidence does not support that the prolonged continuation of the luteal phase support improves the pregnancy rates or eliminates the number of miscarriages [39,40]. Lastly, although segmentation of the cycle and freezing of all embryos is an appropriate alternative for other centres, in Vietnam, this was not financially feasible at the time of the study, with patients having requested a fresh transfer irrespective of the associated risk. Although an alternative modified luteal support strategy of 1.500 IU hCG at oocyte retrieval [9] was previously suggested, this strategy was not used due to the clinicians’ concerns regarding potential OHSS with exposure to even small amounts of exogenous hCG in these OHSS high risk patients.

Additional trials should ascertain whether the combination of GnRHa trigger and the intensive luteal steroid support package in the OHSS high-risk patient is associated with better clinical outcomes than the use of GnRHa trigger and subsequent frozen embryo transfer.

## Conclusions

This large retrospective cohort study confirms that in Asian women who are undergoing ovarian stimulation and develop an excessive ovarian response, the use of a GnRHa trigger combined with intensive luteal steroid support, only, can provide them with the opportunity to proceed to fresh embryo transfer with satisfactory live birth rates. However, there is still a minor chance of late onset severe OHSS development despite the absence of exogenous HCG supplementation. RCTs are warranted to provide upper cut off limits of follicular response which will determine when fresh embryo transfer with GnRHa trigger should be avoided.

## Abbreviations

OHSS: Ovarian hyperstimulation syndrome; GnRHa: GnRH agonist; IVF: In-vitro fertilisation; E2: oestradiol; SD: Standard deviation; CI: Confidence intervals.

## Competing interests

The authors declare that they have no competing interests.

## Authors’ contributions

SMN and PH conceived the study. VTNL, HMT and PHT provided the original data. SI collated the data and performed the statistical analysis. SI, SMN and PH wrote the first draft of the manuscript. All co-authors contributed to the final manuscript. All authors read and approved the final manuscript.

## Authors information

Peter Humaidan, Scott M Nelson, Joint senior authors.

## References

[B1] Itskovitz J Erlik Y Boldes R Kahana L Levron J Brandes JM Induction of preovulatory luteinizing-hormone surge and prevention of ovarian hyperstimulation syndrome by gonadotropin-releasing-hormone agonist Fertil Steril 1991 6 213 220 1906406

[B2] Itskovitz-Eldor J Kol S Mannaerts B Use of a single bolus of GnRH agonist triptorelin to trigger ovulation after GnRH antagonist ganirelix treatment in women undergoing ovarian stimulation for assisted reproduction, with special reference to the prevention of ovarian hyperstimulation syndrome: preliminary report: short communication Hum Reprod 2000 6 1965 1968 10.1093/humrep/15.9.1965 10966996

[B3] Hoff JD Quigley ME Yen SSC Hormonal dynamics at midcycle- a reevaluation J Clin Endocrinol Metab 1983 6 792 796 10.1210/jcem-57-4-792 6411753

[B4] Beckers NG Macklon NS Eijkemans MJ Ludwig M Felberbaum RE Diedrich K Bustion S Loumaye E Fauser BC Nonsupplemented luteal phase characteristics after the administration of recombinant human chorionic gonadotropin, recombinant luteinizing hormone, or gonadotropin-releasing hormone (GnRH) agonist to induce final oocyte maturation in in vitro fertilization patients after ovarian stimulation with recombinant follicle-stimulating hormone and GnRH antagonist cotreatment J Clin Endocrinol Metab 2003 6 4186 4192 10.1210/jc.2002-021953 12970285

[B5] Kolibianakis EM Schultze-Mosgau A Schroer A van Steirteghem A Devroey P Diedrich K Griesinger G A lower ongoing pregnancy rate can be expected when GnRH agonist is used for triggering final oocyte maturation instead of HCG in patients undergoing IVF with GnRH antagonists Hum Reprod 2005 6 2887 2892 10.1093/humrep/dei150 15979994

[B6] Humaidan P Bredkjaer HE Bungum L Bungum M Grondahl ML Westergaard L Andersen CY GnRH agonist (buserelin) or hCG for ovulation induction in GnRH antagonist IVF/ICSI cycles: a prospective randomized study Hum Reprod 2005 6 1213 1220 10.1093/humrep/deh765 15760966

[B7] Pirard C Donnez J Loumaye E GnRH agonist as luteal phase support in assisted reproduction technique cycles: results of a pilot study Hum Reprod 2006 6 1894 1900 10.1093/humrep/del072 16556673

[B8] Humaidan P Bungum L Bungum M Andersen CY Rescue of corpus luteum function with peri-ovulatory HCG supplementation in IVF/ICSI GnRH antagonist cycles in which ovulation was triggered with a GnRH agonist: a pilot study Reprod Biomed Online 2006 6 173 178 10.1016/S1472-6483(10)60612-8 16895629

[B9] Humaidan P Bredkjaer HE Westergaard LG Andersen CY 1,500 IU human chorionic gonadotropin administered at oocyte retrieval rescues the luteal phase when gonadotropin-releasing hormone agonist is used for ovulation induction: a prospective, randomized, controlled study Fertil Steril 2010 6 847 854 10.1016/j.fertnstert.2008.12.042 19200959

[B10] Humaidan P Luteal phase rescue in high-risk OHSS patients by GnRHa triggering in combination with low-dose HCG: a pilot study Reprod Biomed Online 2009 6 630 634 10.1016/S1472-6483(10)60006-5 19549440

[B11] Humaidan P Polyzos NP Alsbjerg B Erb K Mikkelsen AL Elbaek HO Papanikolaou EG Andersen CY GnRHa trigger and individualized luteal phase hCG support according to ovarian response to stimulation: two prospective randomized controlled multi-centre studies in IVF patients Hum Reprod 2013 6 2511 2521 10.1093/humrep/det249 23753114

[B12] Babayof R Margalioth EJ Huleihel M Amash A Zylber-Haran E Gal M Brooks B Mimoni T Eldar-Geva T Serum inhibin A, VEGF and TNF alpha levels after triggering oocyte maturation with GnRH agonist compared with HCG in women with polycystic ovaries undergoing IVF treatment: a prospective randomized trial Hum Reprod 2006 6 1260 1265 10.1093/humrep/dei475 16439507

[B13] Engmann L DiLuigi A Schmidt D Nulsen J Maier D Benadiva C The use of gonadotropin-releasing hormone (GnRH) agonist to induce oocyte maturation after cotreatment with GnRH antagonist in high-risk patients undergoing in vitro fertilization prevents the risk of ovarian hyperstimulation syndrome: a prospective randomized controlled study Fertil Steril 2008 6 84 91 10.1016/j.fertnstert.2007.02.002 17462639

[B14] Shapiro BS Daneshmand ST Garner FC Aguirre M Hudson C Comparison of “triggers” using leuprolide acetate alone or in combination with low-dose human chorionic gonadotropin Fertil Steril 2011 6 2715 2717 10.1016/j.fertnstert.2011.03.109 21550042

[B15] Imbar T Kol S Lossos F Bdolah Y Hurwitz A Haimov-Kochman R Reproductive outcome of fresh or frozen-thawed embryo transfer is similar in high-risk patients for ovarian hyperstimulation syndrome using GnRH agonist for final oocyte maturation and intensive luteal support Hum Reprod 2012 6 753 759 10.1093/humrep/der463 22252086

[B16] Orvieto R Intensive luteal-phase support with oestradiol and progesterone after GnRH-agonist triggering: does it help? Reprod Biomed Online 2012 6 680 681 author reply 682–683 10.1016/j.rbmo.2012.03.005 22503268

[B17] Kummer N Benadiva C Feinn R Mann J Nulsen J Engmann L Factors that predict the probability of a successful clinical outcome after induction of oocyte maturation with a gonadotropin-releasing hormone agonist Fertil Steril 2011 6 63 68 10.1016/j.fertnstert.2011.04.050 21565337

[B18] Sunkara SK Rittenberg V Raine-Fenning N Bhattacharya S Zamora J Coomarasamy A Association between the number of eggs and live birth in IVF treatment: an analysis of 400 135 treatment cycles Hum Reprod 2011 6 1768 1774 10.1093/humrep/der106 21558332

[B19] Kolibianakis EM Albano C Kahn J Camus M Tournaye H Van Steirteghem AC Devroey P Exposure to high levels of luteinizing hormone and estradiol in the early follicular phase of gonadotropin-releasing hormone antagonist cycles is associated with a reduced chance of pregnancy Fertil Steril 2003 6 873 880 10.1016/S0015-0282(02)04920-8 12749423

[B20] Humaidan P Quartarolo J Papanikolaou EG Preventing ovarian hyperstimulation syndrome: guidance for the clinician Fertil Steril 2010 6 389 400 10.1016/j.fertnstert.2010.03.028 20416867

[B21] RCOG The management of ovarian hyperstimulation syndrome 2006 London: Royal College of Obstetricians and Gynaecologists

[B22] Griesinger G Diedrich K Devroey P Kolibianakis EM GnRH agonist for triggering final oocyte maturation in the GnRH antagonist ovarian hyperstimulation protocol: a systematic review and meta-analysis Hum Reprod Update 2006 6 159 168 16254001 10.1093/humupd/dmi045

[B23] Youssef MA Van der Veen F Al-Inany HG Griesinger G Mochtar MH Aboulfoutouh I Khattab SM Van Wely M Gonadotropin-releasing hormone agonist versus HCG for oocyte triggering in antagonist assisted reproductive technology cycles Cochrane Database Syst Rev 2011 6 CD008046 21249699 10.1002/14651858.CD008046.pub3

[B24] Homburg R Ray A Bhide P Gudi A Shah A Timms P Grayson K The relationship of serum anti-Mullerian hormone with polycystic ovarian morphology and polycystic ovary syndrome: a prospective cohort study Hum Reprod 2013 6 1077 1083 10.1093/humrep/det015 23377771

[B25] Humaidan P Kol S Papanikolaou EG GnRH agonist for triggering of final oocyte maturation: time for a change of practice? Hum Reprod Update 2011 6 510 524 10.1093/humupd/dmr008 21450755

[B26] Yovich JL Stanger JD Yovich JM Tuvik AI Turner SR Hormonal profiles in the follicular phase, luteal phase and first trimester of pregnancies arising from in-vitro fertilization Br J Obstet Gynaecol 1985 6 374 384 10.1111/j.1471-0528.1985.tb01112.x 2580551

[B27] Van der Linden M Buckingham K Farquhar C Kremer JA Metwally M Luteal phase support for assisted reproduction cycles Cochrane Database Syst Rev 2011 6 CD009154 21975790 10.1002/14651858.CD009154.pub2

[B28] Griffin D Benadiva C Kummer N Budinetz T Nulsen J Engmann L Dual trigger of oocyte maturation with gonadotropin-releasing hormone agonist and low-dose human chorionic gonadotropin to optimize live birth rates in high responders Fertil Steril 2012 6 1316 1320 10.1016/j.fertnstert.2012.03.015 22480822

[B29] Purcell K Schembri M Frazier LM Rall MJ Shen S Croughan M Grainger DA Fujimoto VY Asian ethnicity is associated with reduced pregnancy outcomes after assisted reproductive technology Fertil Steril 2007 6 297 302 10.1016/j.fertnstert.2006.06.031 17081529

[B30] Huddleston HG Rosen MP Lamb JD Modan A Cedars MI Fujimoto VY Asian ethnicity in anonymous oocyte donors is associated with increased estradiol levels but comparable recipient pregnancy rates compared with Caucasians Fertil Steril 2010 6 2059 2063 10.1016/j.fertnstert.2009.11.019 20056204

[B31] Griesinger G Schultz L Bauer T Broessner A Frambach T Kissler S Ovarian hyperstimulation syndrome prevention by gonadotropin-releasing hormone agonist triggering of final oocyte maturation in a gonadotropin-releasing hormone antagonist protocol in combination with a “freeze-all” strategy: a prospective multicentric study Fertil Steril 2011 6 2029 U2195 10.1016/j.fertnstert.2011.01.163 21371705

[B32] Kol S Humaidan P GnRH agonist triggering: recent developments Reprod BioMed Online 2013 6 226 230 10.1016/j.rbmo.2012.11.002 23337420

[B33] Mathur RS Akande AV Keay SD Hung LP Jenkins JM Distinction between early and late ovarian hyperstimulation syndrome Fertil Steril 2000 6 901 907 10.1016/S0015-0282(00)00492-1 10785214

[B34] Lawlor DA Nelson SM Effect of age on decisions about the numbers of embryos to transfer in assisted conception: a prospective study Lancet 2012 6 521 527 10.1016/S0140-6736(11)61267-1 22243709

[B35] Devroey P Polyzos NP Blockeel C An OHSS-Free Clinic by segmentation of IVF treatment Hum Reprod 2011 6 2593 2597 10.1093/humrep/der251 21828116

[B36] Nelson SM Lawlor DA Predicting live birth, preterm delivery, and low birth weight in infants born from in vitro fertilisation: a prospective study of 144,018 treatment cycles PLoS Med 2011 6 e1000386 10.1371/journal.pmed.1000386 21245905 PMC3014925

[B37] Nevo O Eldar-Geva T Kol S Itskovitz-Eldor J Lower levels of inhibin A and pro-alphaC during the luteal phase after triggering oocyte maturation with a gonadotropin-releasing hormone agonist versus human chorionic gonadotropin Fertil Steril 2003 6 5 1123 1128 10.1016/S0015-0282(03)00177-8 12738506

[B38] Papanikolaou EG Humaidan P Polyzos N Kalantaridou S Kol S Benadiva C Tournaye H Tarlatzis B New algorithm for OHSS prevention Reprod Biol Endocrinol 2011 6 147 10.1186/1477-7827-9-147 22054506 PMC3230134

[B39] Nyboe Andersen A Popovic-Todorovic B Schmidt KT Loft A Lindhard A Hojgaard A Ziebe S Hald F Hauge B Toft B Progesterone supplementation during early gestations after IVF or ICSI has no effect on the delivery rates: a randomized controlled trial Hum Reprod 2002 6 357 361 10.1093/humrep/17.2.357 11821278

[B40] Schmidt KL Ziebe S Popovic B Lindhard A Loft A Andersen AN Progesterone supplementation during early gestation after in vitro fertilization has no effect on the delivery rate Fertil Steril 2001 6 337 341 10.1016/S0015-0282(00)01709-X 11172836

